# Origin and Evolution of the *Azolla* Superorganism

**DOI:** 10.3390/plants13152106

**Published:** 2024-07-29

**Authors:** Jonathan Bujak, Alexandra Bujak

**Affiliations:** 1 Azolla Biosystems Ltd., Poulton-Le-Fylde FY6 8JX, UK; 2 The Azolla Foundation, Blackpool FY2 9JS, UK; abujak@theazollafoundation.org

**Keywords:** *Anabaena*, *Azolla*, climate change, cyanobacteria, evolution, genetics, *Nostoc*, sequestration, symbiosis

## Abstract

*Azolla* is the only plant with a co-evolving nitrogen-fixing (diazotrophic) cyanobacterial symbiont (cyanobiont), *Nostoc azollae*, resulting from whole-genome duplication (WGD) 80 million years ago in *Azolla*’s ancestor. Additional genes from the WGD resulted in genetic, biochemical, and morphological changes in the plant that enabled the transmission of the cyanobiont to successive generations via its megaspores. The resulting permanent symbiosis and co-evolution led to the loss, downregulation, or conversion of non-essential genes to pseudogenes in the cyanobiont, changing it from a free-living organism to an obligate symbiont. The upregulation of other genes in the cyanobiont increased its atmospheric dinitrogen fixation and the provision of nitrogen-based products to the plant. As a result, *Azolla* can double its biomass in less than two days free-floating on fresh water and sequester large amounts of atmospheric CO_2_, giving it the potential to mitigate anthropogenic climate change through carbon capture and storage. *Azolla*’s biomass can also provide local, low-cost food, biofertiliser, feed, and biofuel that are urgently needed as our population increases by a billion every twelve years. This paper integrates data from biology, genetics, geology, and palaeontology to identify the location, timing and mechanism for the acquisition of a co-evolving diazotrophic cyanobiont by *Azolla*’s ancestor in the Late Cretaceous (Campanian) of North America.

## 1. Introduction

### 1.1. The Importance of Nitrogen

Nitrogen is essential for all life on Earth. It is one of the five elements that form nucleotides, which are the building blocks of deoxyribonucleic acid (DNA) that contains genetic information and biological instructions for the development and functioning of all organisms. It is a component of proteins and photosynthetic pigments, including chlorophyll, that enable algae and plants to harvest energy from the sun; however, they cannot obtain it from the atmosphere because of the strength of the dinitrogen (N_2_) triple bond. The only organisms that can sever the bond and synthesise dinitrogen are a group of prokaryotes, called diazotrophs, that evolved the process more than 2.7 billion years ago using an enzyme called nitrogenase and a process called biological nitrogen fixation (BNF) [1,2].

**Figure 1:**
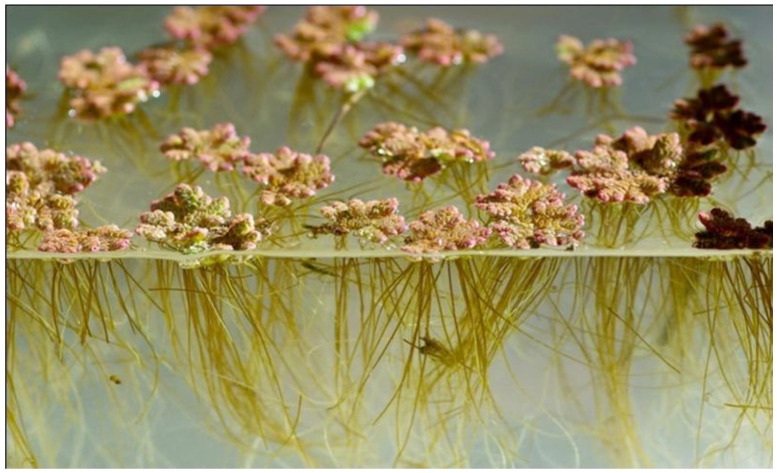
*Azolla filiculoides* showing its floating leaves and root-like tendrils. Photograph from ‘The Azolla Story’ [11].

A few fungi and plants, including some legumes, form temporary symbioses with diazotrophs, but the relationship is severed when the fungus or plant dies and has to be re-established by successive generations. *Azolla* is the only plant with a co-evolving nitrogen-fixing cyanobacterial symbiont (cyanobiont), *Nostoc azollae*, that is passed directly to successive generations of the plant via its megaspores. Colonies of the cyanobacteria live in *Azolla*’s floating leaves, providing nitrogen-based nutrients to the plant that enable it to double its biomass in less than two days free-floating on fresh water [3] (Figure 1). As a result, *Azolla* has been used for hundreds of years in India and the Far East as a renewable, low-cost livestock feed and nitrogen biofertiliser for paddy rice [4]. It also reduces mosquito breeding populations by 95% [5,6,7] and the emissions of the potent greenhouse gas, methane, from paddies by up to 50% [8,9,10] when it covers the water surface in rice paddies.

**Figure 2:**
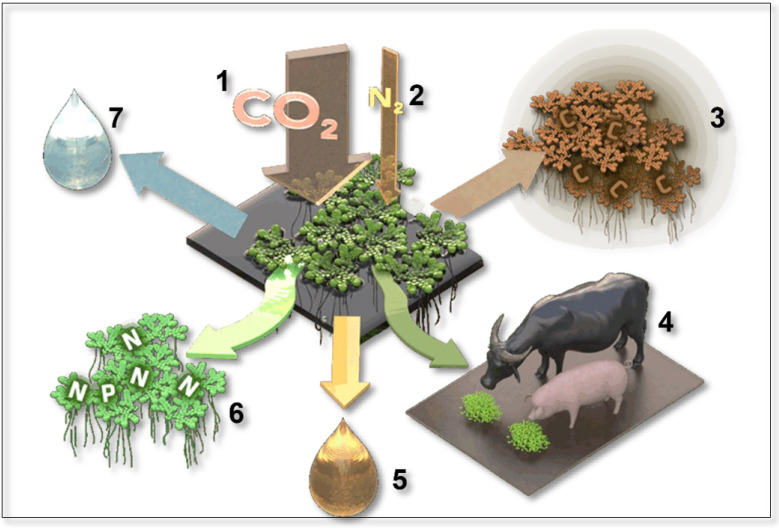
Azolla’s CO_2_ and N_2_ sequestration (1,2) and its products: (3) captured carbon for carbon capture and storage, (4) food and livestock feed, (5) biofuel, (6) biofertiliser, (7) nitrogen-enriched water to fertilise other plants. Illustration rendered by Victor Leshyk (https://www.victorleshyk.com/).

*Azolla* can provide local biofertiliser, livestock feed, biofuel, and food [12] globally when grown indoors [11], helping to alleviate shortages of the ‘three Fs’ that threaten food supplies, as follows: fertiliser, feed, and fuel. It absorbs and removes phosphates and nitrates from water contaminated by chemical fertilisers, industrial pollutants, and animal and human waste that trigger toxic cyanobacterial (also known as blue-green algal) blooms in rivers and lakes. The symbionts’ combined CO_2_ sequestration increases *Azolla*’s carbon capture so that it can sequester large amounts of atmospheric CO_2_, with the plants being compressed and permanently stored to reduce anthropogenic climate change through carbon capture and storage (CCS) (Figure 2). See ‘The Azolla Story’ for a review of its uses [11].

*Azolla* can, therefore, mitigate many of the threats arising from a perfect storm as our population increases by more than a million every three days. Its remarkable properties are increasingly recognised, and it was designated as a unique superorganism by Francisco Carrapiço in 2010 [13], but the origin of *Azolla*’s co-evolving symbiosis with *N. azollae* has, until recently, been obscure. This paper integrates data from biology, genetics, geology, and palaeontology to identify the location, timing, and mechanism of their unique relationship.

### 1.2. Azolla’s Suprageneric Classification

The name ‘azolla’ was introduced by the French naturalist Jean-Baptiste Lamarck in his 1783 publication, *Encyclopédie Méthodique*, on plants collected from South America by the naturalist Philibert Commerson and his assistant Jeanne Baret (also known as Baré, Barret) during Louis-Antoine de Bougainville’s 1767–1768 circumnavigation of the world [14]. *Azolla*’s suprageneric classification varies, with some authors assigning *Azolla* to the monogeneric Family Azollaceae [15] and others assigning the genera *Azolla* and *Salvinia* to the free-floating family Salviniaceae, although *Azolla* can also root in damp soil or mud [16,17,18]. The family Salviniaceae is assigned to the order Salviniales, also known as water ferns, which includes the family Marsileaceae, comprising three genera (*Marsilea*, *Pilularia*, *Regnelidium*) that root in mud and are not free-floating.

### 1.3. The Cyanobacterial Symbiont

**Figure 3:**
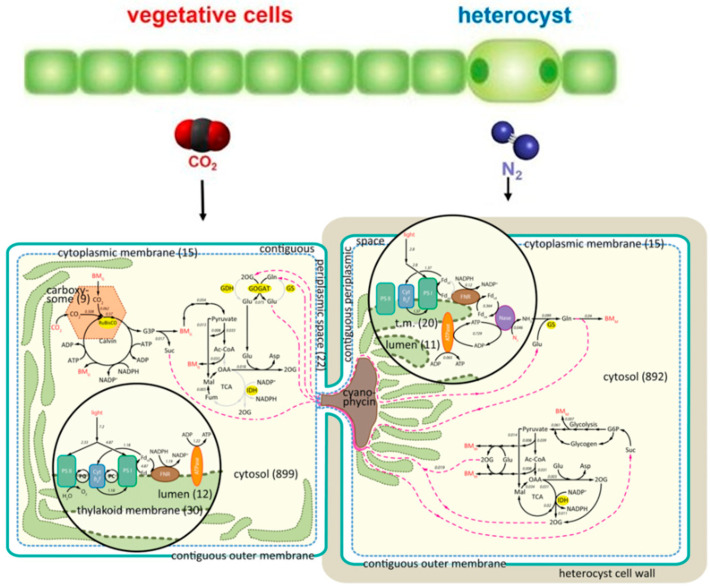
*Nostoc azollae*’s vegetative cells and heterocysts showing the complex biochemical pathways used for photosynthesis and nitrogen fixation. Cyanophycin acts as a reservoir for fixed nitrogen in the heterocysts and enables transport of fixed nitrogen to adjacent vegetative cells. Details of *N. azollae*’s cells from Malatinszky et al. (2017) [20].

*Azolla*’s cyanobiont has been assigned to both *Anabaena azollae* and *Nostoc azollae* because it resembles the free-living species of both genera by having chains of cells (filaments) comprising photosynthetic vegetative cells and thicker-walled heterocysts containing the nitrogen-fixing enzyme nitrogenase that is destroyed by oxygen (Figure 3). Akinetes (resting cells) may also be present, as their germination ensures survival during stressed conditions [19]. We use the name *Nostoc azollae* for consistency with other papers in PLANT’S Special Issue ‘Plant–Cyanobacteria Symbiosis: From Morphology to Practical Uses’.

*N. azollae*’s filaments are immobilised in a gelatinous mucilage sheath around the periphery of a chamber inside *Azolla*’s dorsal floating leaves, with pores permitting the exchange of air, but not microorganisms, including prokaryotes, with the chamber. Slender rods called trichomes transport chemicals and nutrients between the cells of *Azolla* and *N. azollae*, with the latter releasing ovoidal capsules called membrane vesicles (MVs) that contain genetic material [21], so that *Azolla* and *N. azollae* are in biochemical and genetic communication.

The vegetative cells of *N. azollae* use photosynthesis to draw down atmospheric CO_2_ and produce organic compounds that they utilise and pass to the heterocysts for their metabolism. The heterocysts fix atmospheric dinitrogen and synthesise it into nitrogen-based compounds that they utilise and pass to the vegetative cells. This involves complex biochemical processes in both types of cells and the presence of cyanophycin at their interface [20] (Figure 3). Cyanophycin, abbreviated from cyanophycin granule peptide (CGP), is a nitrogen polymer that acts as a reservoir for fixed nitrogen in the heterocysts, enables the transport of fixed nitrogen to adjacent vegetative cells, and inhibits the transport of soluble nitrogen products from vegetative cells to heterocysts [22].

The differentiation of heterocysts and vegetative cells probably evolved in *Anabaena*’s and *Nostoc*’s common ancestor during the Great Oxidation Event (GOE) between 2.5 and 2.1 billion years ago, when the Earth’s atmosphere was enriched in oxygen, resulting in the mass extinction of many anaerobic prokaryotes [23,24,25]. The heterocysts enclosed an oxygen-free environment containing nitrogenase, with cyanophycin providing a selective barrier and conduit between the heterocysts and oxygenated vegetative cells. As a result of these ancient adaptations, today’s species of *Anabaena* and *Nostoc*, including *Azolla*’s cyanobiont *N. azollae*, are the most biochemically and morphologically complex multicellular cyanobacteria with differentiated akinetes, heterocysts, and vegetative cells.

### 1.4. Azolla’s Transmission of N. azollae via Its Megaspores

*Azolla* is unique in its intergenerational transmission of a diazotroph via its spores. This involves biochemical and morphological processes unknown in any other plant, which resulted from *Azolla*’s adaptation and utilisation of morphological changes that occur in free-living *Anabaena* and *Nostoc*. These enable *N. azollae*’s movement inside *Azolla* and its fusion with *Azolla*’s female megaspores during their dormancy and germination into a new plant.

Some free-living species of *Anabaena* and *Nostoc* change their morphology, mobility, and nitrogen fixation in response to changes in the environment and the emission of chemicals emitted by several plants, with which they have a temporary symbiosis [26,27,28], as follows:

- Sessile filaments have vegetative cells that sequester carbon dioxide and heterocysts that fix atmospheric nitrogen. Akinetes may also be present;
- Motile hormogonia only have vegetative cells, so they cannot sequester nitrogen. Hormogonia are produced by free-living species of *Anabaena* and *Nostoc*, including those that have temporary symbioses with plants like *Gunnera manicata*. The plants emit hormogonium-inducing factor (HIF) to induce *Nostoc* in the soil to change into hormogonia that infect specialised stem glands in the plant, after which they change into sessile filaments, including heterocysts, that provide nitrogen-based compounds to the plant;
- Akinetes, or resting spores, are produced by free-living *Anabaena* and *Nostoc*, enabling them to survive under adverse conditions.

The ways in which *Azolla* adapted these processes inside the plant are unique and involved genetic, biochemical, and morphological adaptations, with a combination of biology, genetics, geology, and palaeontology identifying the location, timing, and mechanism in *Azolla*’s ancestor.

## 2. Evidence for *Azolla*’s Origin

### 2.1. Genetic Evidence for Azolla’s Origin

**Figure 4:**
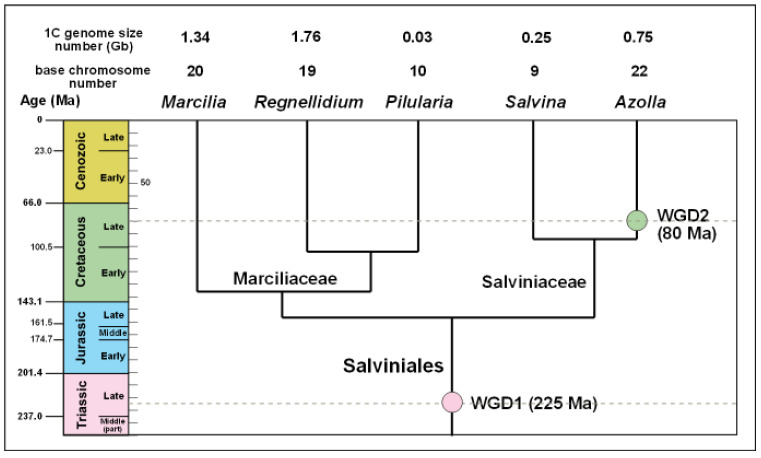
Genome size of the order Salviniales (water ferns), divergence times and two whole-genome duplication (WGD) events of the Salviniales based on phylogenetic mapping. Geological scale on the left is in millions of years. Figure redrawn and expanded from Li et al. (2018) [17].

The phylogeny and genetic events shown in Figure 4 are crucial for understanding *Azolla*’s unique relationship with *N. azollae* because they document the key genetic event that resulted in their fusion into a unique superorganism—a process called whole-genome duplication (WGD). WGDs are important evolutionary events because they ‘provide a source of genetic material for mutation, drift, and selection to act upon, making new evolutionary opportunities possible’ [29] (Abstract).

Li et al. (2018) identified the following two WGD events in Salviniales based on genetic analysis: an earlier event (WGD1 in Figure 4) 225 million years ago in the Late Triassic and a later event (WGD2) 80 million years ago in the Late Cretaceous [17]. WGD1 may have facilitated the Late Triassic colonisation of aquatic habitats by Salviniales, after which they evolved into two families, Marsileaceae and Salviniaceae, near the end of the Jurassic about 155 million years ago. Salviniaceae then diverged into the following two lineages, each having one extant genus: *Azolla*, which has a co-evolving nitrogen-fixing cyanobacterium, and *Salvinia*, which does not possess one. Their difference is indicated by the relative size of *Azolla* and *Salvinia*’s genomes, with *Azolla*’s being approximately three times larger than that of *Salvinia*, with more than twice the number of chromosomes (Figure 4). They differ in size because *Azolla*’s ancestor underwent WGD about 80 million years ago *after* the two genera diverged from a common ancestor. The 80 million-year-old WGD event, therefore, only affected *Azolla*. It did not affect any other plant, and that is one of the reasons why *Azolla* is unique.

*N. azollae*’s co-evolution with *Azolla* resulted in extensive changes to the cyanobiont’s genome compared to the free-living species of *Anabaena* and *Nostoc* [30,31,32,33]. These alterations involved the downregulation, loss, or conversion to pseudogenes of some genes, changing *N. azollae*’s ancestors from independent free-living organisms into ones that could not survive outside *Azolla*. The upregulation of other genes enhanced *N. azollae*’s sequestration of atmospheric nitrogen and the provision of nitrogen-based compounds to *Azolla*, increasing the plant’s speed of growth free-floating on fresh water.

Approximately one-third of *N. azollae*’s genome comprises non-functional pseudogenes. These include pseudogenes originating from genes that had previously expressed proteins involved in filament growth and division, reflecting the need to restrict their number in the plant’s confined leaf cavities. Other pseudogenes reflect a loss of function relating to the cyanobiont’s assimilation of nitrogen, so that increased amounts of nitrogen-based compounds are transferred from the cyanobiont’s cells to those of *Azolla*, facilitating the plant’s growth and its provision of almost three times more nitrogen biofertiliser than other plants, including legumes [34].

A third set of pseudogenes originated from genes that previously expressed proteins involved in the synthesis of carotenoid and chlorophyll pigments. Larsson et al. (2011) analysed the genomes of 57 free-living cyanobacteria and *N. azollae* for shared and unique orthologs and found that two protein groups occur in the genomes of all the cyanobacteria, except *N. azollae*, in which they are represented by non-functional pseudogenes [32]. These correspond to (1) geranylgeranyl pyrophosphate synthase, which is involved in the synthesis of carotenoids and chlorophyll, and (2) uroporphyrinogen-III synthase HemD, which generates precursors of tetrapyrroles that provide protection against photooxidative damage. *N. azollae* is, therefore, reliant on *Azolla*’s cellular pigments for protection against photooxidative damage, so it cannot survive outside the plant. The previously independent cyanobacterium became an obligate symbiont and an indispensable partner of the *Azolla* superorganism. It also resulted in the complementary photosynthesis of *Azolla* and its cyanobiont, with *Azolla* utilising shorter wavelength pigments, including chlorophyll a, chlorophyll b, and β-carotene, and *N. azollae* utilising longer wavelength pigments, including phycoerythrin and phycocyanin. As a result, *Azolla* is a shade plant that needs little illumination for its growth and proliferation, facilitating its growth next to rice plants in paddies and its cultivation for CCS and renewable food, biofertiliser, livestock feed, and biofuel.

In contrast to *N. azollae*’s pseudogenes, genes that were retained or up-regulated in *N. azollae* enhance its symbiosis with *Azolla* and, especially, the sequestration and synthesis of atmospheric nitrogen, reflecting *N. azollae*’s primary symbiotic role in providing nitrogen-based compounds to *Azolla*. The entire set of genes related to nitrogen fixation (the nif gene cluster) is, therefore, intact in *N. azollae* because those genes express proteins involved in the conversion of atmospheric nitrogen into nitrogen-based compounds.

*N. azollae*’s provision of nitrogen-based compounds is also increased by the number of heterocysts that assimilate atmospheric nitrogen. All twenty-two genes related to heterocyst formation are intact in *N. azollae*, but the pats gene, which expresses proteins involved in the suppression of heterocyst development, is absent. *N. azollae*, therefore, produces more heterocysts than free-living cyanobacteria, resulting in chains of cells that contain up to 30% heterocysts compared to less than 10% in free-living *Anabaena* and *Nostoc*. This enables *N. azollae* to fix between four and eighteen times more atmospheric nitrogen than other species of *Anabaena* and *Nostoc* [33,35]. As a result, *Azolla* provides more nitrogen biofertiliser than any other plant, increasing its potential to augment or replace nitrogen-based chemical fertilisers.

### 2.2. Palaeontological Evidence for Azolla’s Origin

A database compiled by the Geological Survey of Canada listed more than 120,000 published species of fossil pollen and spores, including 89 species of the genus *Azolla* and several species of the similar genera *Azollopsis*, *Paleoazolla*, and *Parazolla* [36]. Subsequent publications were assessed in the present study, including those documenting the Eocene Arctic Azolla Event that triggered the initial shift from a greenhouse to an icehouse climate 49 million years ago [37,38].

**Figure 5:**
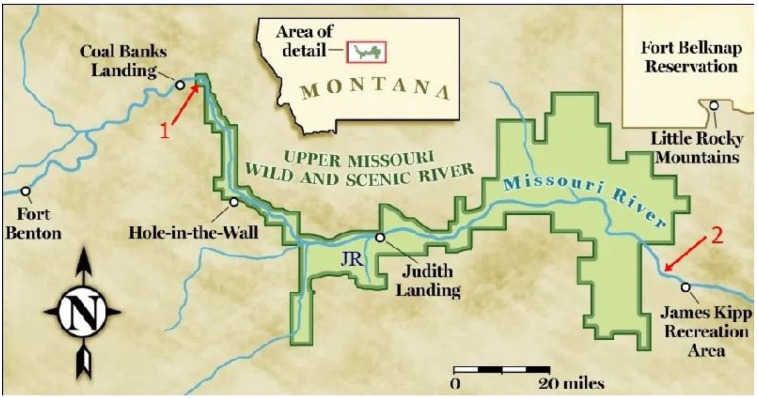
Location of rocks sampled by Hall (1969) [39] containing (1) *Parazolla heterotricha* and (2) *Azolla simplex*. UMRBNM is shown in green. JR = Judith River. Map modified from Scouting Magazine [51].

The oldest confident record of *Azolla* is that of Hall (1969) from Campanian rocks of the western interior of North America [39] (Figure 5). Reported occurrences of *Azolla* and *Azollopsis* from older strata are questionable because of the misidentification of specimens or erroneous assignments of their age from the Late Cretaceous (Turonian-Cenomanian) of Kazakhstan [40,41] and India [42] and the Jurassic to Early Cretaceous of India [43], Kazakhstan [44], Tajikistan [40], Turkmenistan [45], Ukraine [46], and eastern Russia [44,47,48,49,50] (Appendix A).

**Figure 6:**
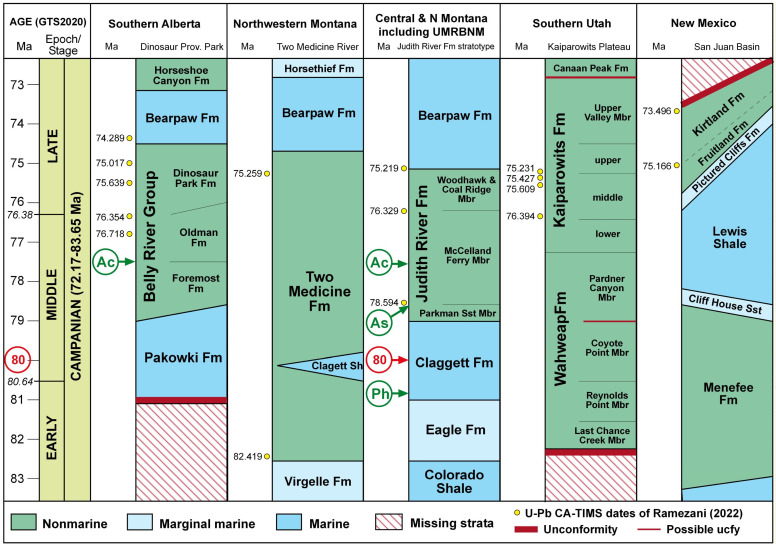
Absolute ages determined for strata in Alberta, Montana, Utah, and New Mexico showing the earliest recorded occurrences of *Azolla simplex* (As), *Parazolla heterotricha* (Ph), and *Azollopsis coccoides* (Ac). Small yellow circles show radiometric dates based on 40Ar/39Ar isotopes. The 80 Ma age indicated by genome analysis for the oldest *Azolla* is circled in red. Figure modified from Ramezani et al. (2022) [54] to include details of taxon occurrences and absolute ages.

Hall (1969) analysed samples from the Claggett and Judith River Formations near the Upper Missouri River Breaks National Monument (UMRBNM) in north-central Montana [39]. Both formations are Campanian, with the Claggett being older, so that together, they potentially preserve a fossil record of *Azolla*’s early development (Figure 6). Hall considered fossils from the Judith River Formation to be the oldest species of *Azolla*, which he named *Azolla simplex*, and he assigned fossils from the Claggett Formation to a new genus and species, *Parazolla heterotricha*.

Hall did not observe the vegetative remains of either species, but the fossilised spores and sporangia of both have distinctive structures called floats and massulae, with glochidia that enable the spores to reach and remain on the water’s surface after germination. Extant species of *Azolla* and *A. simplex* have distinctive floats and anchor-shaped glochidia, whereas those of *P. heterotricha* are hair-like, which, according to Hall, are more ‘primitive’ [39]. He, therefore, proposed that *P. heterotricha* was the ancestor of *A. simplex* based on their morphology and relative ages and that *A. simplex* was the oldest species of *Azolla*.

As discussed earlier, *Azolla* is unique in having a permanent diazotrophic cyanobiont that is transmitted to subsequent generations of the plant by its megaspores. It is presently uncertain whether *P. heterotricha* also had a temporary or permanent diazotrophic cyanobiont. We, therefore, use the informal term ‘azolla’ in the following text for the earliest plant that had a permanent diazotrophic cyanobiont and the term ‘proto-azolla’ for its immediate ancestor that had a temporary diazotrophic cyanobiont.

### 2.3. Age of the Oldest Azolla Fossils

The Campanian Stage extends from 83.7 to 72.2 million years (Ma) based on Gradstein et al.’s (2020) Geological Time Scale [52], with the succeeding Maastrichtian Stage extending up to 66.04 Ma, co-eval with the end-Cretaceous Yucatan bolide impact. Rogers (2016) determined the age in millions of years of three horizons in the Judith River Formation using the radiometric dating of ^40^Ar/^39^Ar argon isotopes in volcanic ash layers called bentonites [53]. The ashes were ejected from volcanoes in the Elkhorn Mountains of southwestern Montana and deposited across freshwater lakes and rivers, including Montana’s UMRBNM and the stratotype of the Judith River Formation.

Additional isotope data from Ramezani et al. (2022) provided ages for relevant sections in Alberta, Mexico, Montana, and Utah [54], including the earliest recorded occurrences of *A. simplex*, *P. heterotrycha*, and the genus *Azollopsis*, as shown in Figure 6.

Fossils of *P. heterotrycha* occur near the base of the Claggett Formation ‘not far above the contact with the Eagle Sandstone’ [now the Eagle Formation] at the western end of the UMRBNM [39] (p. 1177). There is no radiometric age assignment for the horizon, but regional correlations indicate that the base of the Claggett Formation is Campanian and no older than 83.7 Ma. Rocks containing *P. heterotrycha* have an age close to 80 Ma based on their thickness and a sedimentation rate of approximately 3.8 cm/1000 years [53]. This is consistent with 80 Ma indicated by genetic analysis for the WGD event that resulted in the earliest *Azolla* [17].

### 2.4. The Geological Evidence

**Figure 7:**
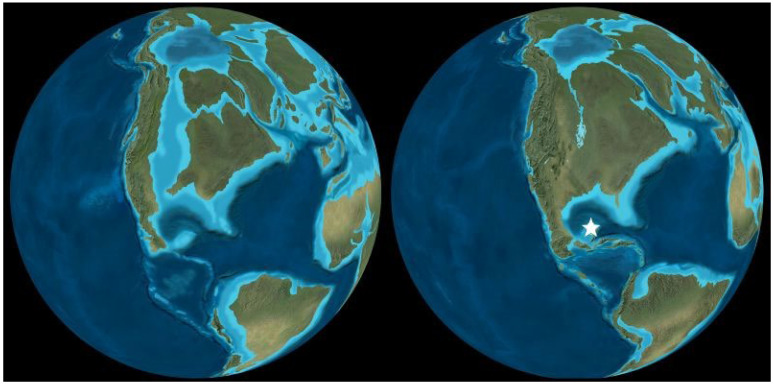
North American palaeogeography at the beginning of the Campanian (83.7 Ma, left) and the end of the Maastrichtian (66.04 Ma, right). Star shows the location of the Yucatan bolide impact. Reconstructions from Deep Time Maps™ [55] ©2020 Colorado Plateau Geosystems, Inc.

The Cretaceous (143.1–66.04 Ma) had a greenhouse climate with polar temperatures that rarely fell below freezing. At the beginning of the Campanian (83.65 Ma), the North American continent was separated by shallow seas into three large islands, with the western isle forming a long narrow landmass extending from today’s Alaska to Mexico [55]. Its western shore bordered the Pacific Ocean, and its eastern coast was lapped by a shallow Western Interior Seaway (WIS) that connected the Arctic Ocean and Gulf of Mexico (Figure 7). Halfway along its length, an eastern arm of the WIS separated two islands before joining the nascent Labrador Sea between North America and Greenland, with most of southern Europe being submerged under a shallow sea punctuated by subtropical islands.

Uplift during the Campanian-Maastrichtian (83.65–66.04 Ma) converted the North American seaways into freshwater rivers, lakes, and nutrient-rich land that had been submerged. Previously separated terrestrial fauna, including dinosaurs like *Tyrannosaurus*, met for the first time and vied for dominance as vegetation rapidly spread across the newly emergent land, including angiosperms, horsetails, cycads, and conifers [56]. Beneath the trees, the undergrowth was filled with a dense cover of ferns, some growing on the edges of ponds and lakes, where they rooted in the shallow substrate. By 80 Ma, during the deposition of the Claggett Formation, these included ‘proto-azolla’ that floated on the surfaces of lakes in today’s Montana.

## 3. Evolution of the Earliest Azolla

### 3.1. Temporary Symbiosis in Azolla’s Ancestor

The integration of data from the past and present provides a model of events that resulted in the evolution of proto-azolla into azolla. Many of today’s freshwater cyanobacterial blooms are triggered by the runoff of phosphate and nitrogen fertilisers [57], and it is probable that the floating leaves of proto-azolla were surrounded by masses of cyanobacteria that thrived on nutrients, and especially phosphates, transported by streams and rivers from newly emergent shales, muds, and fertile soils.

Today’s temporary symbioses with diazotrophs occur in several plants and fungi, including the hornwort *Anthoceros punctatus*, the liverwort *Blasia*, the angiosperm *Gunnera*, some legumes, and cycads [58,59,60]. The most common are legumes that temporarily house nitrogen-fixing soil bacteria called rhizobia in their root nodules, indicating that *N. azollae*’s presence in the floating leaves of extant *Azolla* resulted from proto-azolla’s leaves being surrounded by floating cyanobacteria that may have occurred as gelatinous masses, like those formed by today’s *Nostoc* [61].

*Gunnera*’s symbiosis is similar to that of *Azolla*, as it involves *Nostoc*. It only occurs when the soil is deficient in nitrogen and begins when the plant emits chemicals called hormogonium-inducing factor (HIF) that induce the cyanobacteria to change from sessile filaments into motile hormogonia [62]. The movement of many prokaryotic hormogonia, including *Nostoc punctiforme*, involves twitching motility through extension–retraction cycles of type IV pili (T4P) augmented by surface waves generated by the contraction of a fibril layer, with *Nostoc* hormogonia achieving speeds of ten micrometres per second [63,64,65,66]. This enables the hormogonia to propel themselves to *Gunnera*’s stems, where they enter specialised glands that are only developed when the plants are deprived of combined nitrogen [62].

The symbiosis, therefore, is promoted by the plant’s nitrogen deficiency, confirming the cyanobacteria’s primary symbiotic role to provide nitrogen to the plant, as seen in today’s species of *Azolla* and, by inference, proto-azolla. During periods of nitrogen deficiency, proto-azolla emitted HIF to trigger a change in the cyanobacteria from sessile filaments into motile hormogonia that propelled themselves towards the floating leaves, where the symbiosis occurred in cavities that developed due to a low combined nitrogen content in the water. Their entry into the cavities is indicated by *Gunnera*’s relationship with *Nostoc*, in which the hormogonia follow each other in orderly lines towards the stem glands [62].

Temporary openings in proto-azolla’s leaves enabled the entry of the cyanobacteria into cavities, where they changed into sessile filaments with nitrogen-fixing heterocysts. This may have been facilitated by the plant’s emission of hormogonium repressing factors (HRF) that are produced by several fungi and plants [28,67,68]. These include *Azolla pinnata*, in which HRFs are more common in the plant’s mature leaves than at the stem apex, providing a reservoir of hormogonia for conversion to filaments in the developing leaves [69].

A temporary symbiosis was, therefore, established in proto-azolla, with entry into its leaf cavity being sealed. The cyanobacteria’s heterocysts provided the plant with nitrogen-based nutrients that promoted its growth, and the plant supplied the cyanobacteria with metabolites. Photosynthesis by cyanobacteria’s vegetative cells was facilitated by light penetrating the plant’s floating leaves, with narrow pores enabling air exchange with the atmosphere. The temporary symbiosis ended when the plant died and had to be re-established by successive generations of proto-azolla.

### 3.2. Whole-Genome Duplication (WGD)

Whole-genome duplication (WGD) occurred in a single plant approximately 80 million years ago, with the additional genes enabling its descendants to transmit the cyanobacteria via their megaspores. This was achieved in the following three ways:

1. Chemical compounds were secreted inside the plant to induce changes in cyanobacterial mode involving motile hormogonia, sessile filaments, and resting akinetes, facilitating their movement, retention, and viability during the plant’s sexual reproduction. Similar chemical and environmental triggers induce the same changes in today’s free-living *Anabaena* and *Nostoc*;
2. A series of passages inside the plant enabled the cyanobacteria to travel from the plant’s leaf cavities to a chamber called the indusium next to its developing megaspore. After the germination of the megaspore, a channel opened in the embryonic leaf (cotyledon), enabling the cyanobacteria to travel from the indusium to the dorsal leaf cavities as they developed in the new plant;
3. The cyanobionts’ movement through the passages was directed by chemicals secreted inside the plant, which were augmented by a negative nitrogen gradient, similar to the attraction of today’s *Nostoc* in the soil to *Gunnera*’s stem nodules.

**Figure 8:**
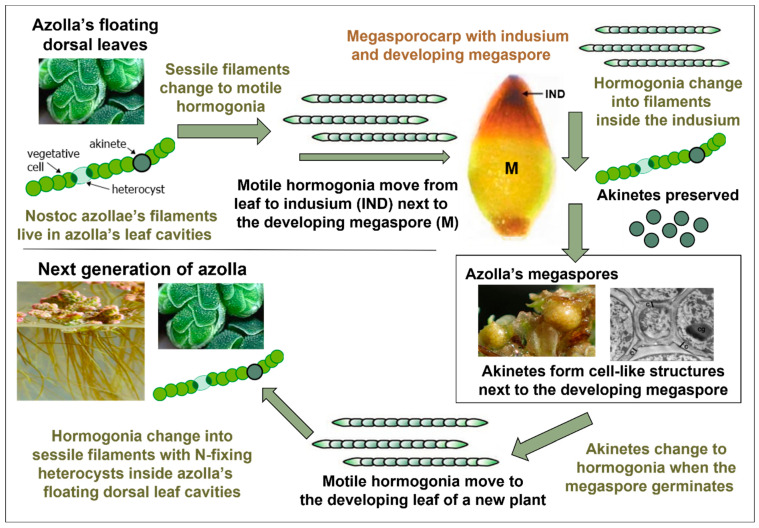
*Azolla*’s intergenerational transmission of *Nostoc azollae* via its megaspores. See text for details.

*Azolla*’s sexual reproduction illustrates the complex strategies and processes that *Azolla*’s and *N. azollae*’s ancestors evolved, enabling them to remain together and co-evolve for 80 million years (Figure 8). *Azolla* produces the following two types of spores during sexual reproduction in structures called sporocarps: (1) female megaspores that occur in conical megasporocarps about half a millimetre in length and (2) male microspores that occur in larger, spherical microsporocarps. Each megasporocarp produces 32 female megaspores, but only one survives inside the megasporocarp, which has a cavity called the indusium inside its narrow end (Figure 8).

Prior to *Azolla’s* sexual reproduction, sessile filaments of *N. azollae* are immobilised in mucilage that fills the periphery of *Azolla’s* floating-leaf cavities, similar to gelatinous masses surrounding free-living *Nostoc* [61]. When *Azolla’s* spores begin to develop, *Azolla* secretes HIF into its leaf cavities, inducing *N. azollae* to change into motile hormogonia in the same way that some species of *Gunnera* secrete HIF outside the plant to induce free-living *Nostoc* to convert to hormogonia and enter the plant’s stem glands. Other chemicals are emitted that dissolve or disaggregate the mucilage, freeing the hormogonia to move inside the plant.

**Figure 9:**
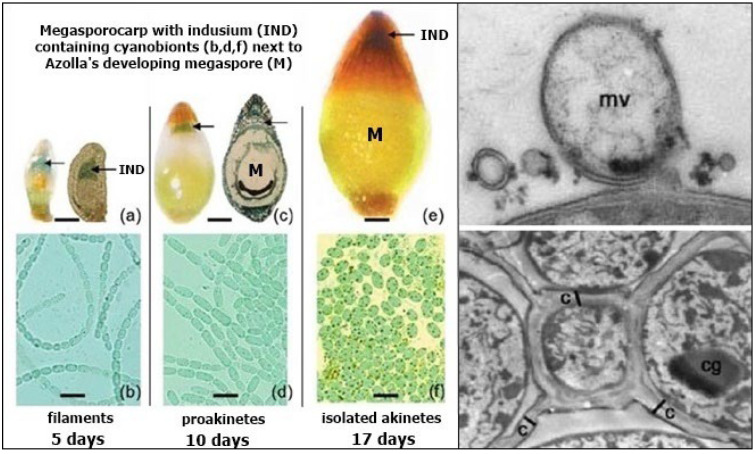
Left: *Azolla*’s megasporocarp (**a**,**c**,**e**) colonised by *N. azollae* in the indusium (IND) next to *Azolla*’s developing megaspore (M), showing *N. azollae*’s change from filaments (**b**) to proakinetes (**d**) to isolated akinetes (**f**). Upper right: Membrane vesicles (mv) released from *N. azollae* contain genetic material. Lower right: Isolated akinetes are immobilised inside a structured biofilm that fills the indusium so that they resemble cells separated by channels (**c**). Cyanophycin granules (cg) in the immobilised akinetes provide reserves of nitrogen, carbon, and energy, facilitating their dormancy, survival, and germination at the same time as *Azolla*’s megaspore. Illustrations modified from Zheng et al. (2009) [70].

*N. azollae*’s hormogonia enter a passage that opens at one end of the leaf’s cavity, providing a conduit for them to travel to *Azolla*’s megasporocarp on the lower, submerged lobe of the leaf, where they enter a cavity called the indusium that encloses them next to the developing megaspore (Figure 9). The hormogonia change back into filaments that excrete membrane vesicles containing genetic material, including DNA, that they exchange with *Azolla* [70]. The filaments of vegetative cells then change into chains of proakinetes before separating into unicellular akinetes that are immobilised by a structured biofilm filling the indusium, with the network of channels and akinetes resembling cells that are fused to the megaspore (Figure 9). Akinetes are unknown inside any plant, except for *Azolla*, and their resistance to adverse conditions enables their survival next to *Azolla*’s megaspore as it develops and germinates into a new plant [70].

### 3.3. Germination and Propagation

*Azolla*’s male microsporocarps are larger than its female megasporocarps and have an indusium chamber that does not contain *N. azollae*, so the cyanobacteria are always transmitted via *Azolla*’s female reproductive organs. Each microsporocarp produces eight motile antherozoids (male gametes) that have flagella, enabling them to approach and fertilise the oosphere (female gamete) produced by the megaspore. This is facilitated by the microsporocarp’s excretion of loose masses of organic material (massulae) that enclose the microspores and antherozoids. The massulae trap air to aid their floatation and extrude anchor-shaped protuberances called glochidia that hook onto the megaspore and facilitate its fertilisation.

Megasporocarp floats that emerge from beneath its indusium cap enable the fertilised oosphere to float to the water’s surface, where it develops into a seedling. This begins with the formation of an embryonic leaf (cotyledon), after which the young plant develops a root, detaches from the megasporocarp, and floats on the water’s surface.

The final stage in *N. azollae*’s transmission to the next generation of the plant is its entry and enclosure in *Azolla’s* developing leaf cavities, as described by Becking (1987) [21]. This is enabled by the conversion of *N. azollae* into motile hormogonia that move into developing cavities of the dorsal leaves, where they change into sessile filaments with photosynthetic vegetative cells and nitrogen-fixing heterocysts. The cycle has now been completed. *N. azollae* has been transmitted to a new generation of *Azolla* via its female megaspores, enabling their co-evolution for approximately 80 million years into a unique superorganism.

## 4. Identifying *Azolla*’s Ancestor

Hall (1969) recorded *Parazolla heterotricha* just above the base of the Campanian Claggett Formation (approximately 81 Ma) and suggested that it was ancestral to *Azolla* [39]. This is consistent with estimates based on the genetics of the earliest *Azolla* at 80 Ma and radiometric dating of the oldest observed *Azolla* fossils at 78.6 Ma (Figure 6) but needs to be confirmed because confident recognition of the earliest *Azolla* is based on its permanent retention of a nitrogen-fixing cyanobiont. Other candidate precursors of *Azolla* are fossils assigned to *Paleoazolla* and *Parazolla*, but all are geologically younger than *A. simplex* (table 3 in [16]). The oldest is *Azollopsis coccoides* (77.5 Ma), which is 1.1 million years younger than *A. simplex* (Figure 6), with a Cenomanian (100.5–93.9 Ma) record of *Azollopsis polyancyra* from South America being re-assigned to the Maastrichtian (72.17–66.04 Ma) [71] (Appendix A).

Detailed sampling of the Claggett and Judith River Formations could provide a record of events that led to the earliest *Azolla*, including the timing and way in which its ancestor developed a permanent symbiosis with *N. azollae*’s ancestor. This depends on the cyanobacteria being transmitted with its megaspores to subsequent generations of the plant, so that the cyanobacteria should occur with megaspores of the earliest *Azolla*, but not with the megaspores of its ancestor. The earliest occurrence of cyanobacteria with *Azolla*’s megaspores, therefore, pinpoints the precise time when the permanent relationship began. Microscopic observation of cyanobacterial filaments or akinetes in the plant’s megasporocarps would confirm their intergenerational symbiosis.

Geochemical analyses could also identify cyanobacterial biomarkers associated with megaspores of the earliest *Azolla*. The presence of 2-methylhopane lipids characterises modern cyanobacteria and has been used to infer their oldest occurrence in sediments deposited close to the GOE at about 2500 Ma [72]. The earliest association of 2-methylhopane biomarkers with *Azolla*’s megaspores, therefore, indicates their transmission to subsequent plant generations and their co-evolution into a unique nitrogen-fixing superorganism. Upper Cretaceous sediments exposed along the banks of the Judith and Missouri rivers in Montana’s UMRBNM may contain this evidence.

## 5. Discussion

*Azolla*’s intergenerational transmission of *N. azollae* via its megaspore is unknown in any other plant and resulted from a WGD event that occurred 80 million years ago in the western interior of North America. Their subsequent co-evolution converted the cyanobiont into a permanent obligate diazotroph unknown in any other plant, enabling *Azolla* to undertake sustainable CCS and provide local renewable food, biofertiliser, livestock feed, and biofuel globally—essentials that are urgently needed by today’s population of more than eight billion people.

## Appendix A. Questionable Pre-Campanian Records of *Azolla* and *Azollopsis*

The oldest confident occurrence of *Azolla* is in Campanian rocks of the western interior of North America [39]. Questionable pre-Campanian occurrences of *Azolla* and *Azollopsis* are listed below from the youngest to oldest age.

*Azolla* sp.: Turonian of Kazakhstan, Central Asia [41]. Review paper without original data for the occurrence of *Azolla* sp.

*Azolla* sp.: Turonian of Kazakhstan, Central Asia [40]. Poor control for the Turonian age assignment.

*Azolla polyancyra*, now *Azollopsis polyancyra* Hall (Sweet and Hills, 1974) [73]: Cenomanian of Argentina [71]. The Cerro Fortaleza Formation that contains *A. polyancyrea* has been re-assigned to the Maastrichtian [74].

*Azolla hamata* Srivastava (1968) [75]: Cenomanian of Andhra Pradesh, India [42]. Data from a subsurface well without cores. *Azolla* probably caved from younger strata.

*Azolla circinata* Hall (1968) [76] and *Azolla pondicherriana* Ramanujam (1996) [43]: Albian of Tamil Nadu, India. Incorrect Albian age assignment. Probable Maastrichtian age.

*Azolla* sp.: Albian of Tajikistan, Central Asia [77]. Review paper without original data for the occurrence of *Azolla* sp.

*Azolla* sp.: Albian of Turkmenistan, Central Asia [45]. Review paper without original data for the occurrence of *Azolla* sp.

*Azolla* sp.: Valanginian of the Zabaykalsky Krai region, eastern Russia [47]. Review paper without original data for the occurrence of *Azolla* sp.

*Azolla* sp.: Valanginian of the Buryatia Republic, south-central Siberia, Russia [50]. Review paper without original data for the occurrence of *Azolla* sp.

*Azolla* sp.: Middle Jurassic of Ukraine [46]. Review paper without original data for the occurrence of *Azolla* sp.

*Azolla* sp.: Middle Jurassic of Kazakhstan, Central Asia [44]. Incorrect identification of specimens assigned to *Azolla*.

*Azolla* sp.: Middle Jurassic of Orenburg Oblast, Eastern Europe, Russia [48]. Incorrect identification of specimens assigned to *Azolla*.

*Azolla* sp. and *Azollopsis* sp.: Early Jurassic to Early Cretaceous. No region specified, probably eastern Russia [49]. Incorrect identification of specimens assigned to *Azolla*.
